# No evidence for effects of Turkish immigrant children‘s bilingualism on executive functions

**DOI:** 10.1371/journal.pone.0209981

**Published:** 2019-01-17

**Authors:** Nils Jaekel, Julia Jaekel, Jessica Willard, Birgit Leyendecker

**Affiliations:** 1 Theory and Practice in Teacher Education, University of Tennessee Knoxville, Tennessee, United States of America; 2 Department of Child and Family Studies, University of Tennessee Knoxville, Tennessee, United States of America; 3 Department of Developmental Psychology, Ruhr-University Bochum, Germany; Nagoya University, JAPAN

## Abstract

Recent research has increasingly questioned the bilingual advantage for executive functions (EF). We used structural equation modeling in a large sample of Turkish immigrant and German monolingual children (N = 337; aged 5–15 years) to test associations between bilingualism and EF. Our data showed no significant group differences between Turkish immigrant and German children’s EF skills while taking into account maternal education, child gender, age, and working memory (i.e., digit span backwards). Moreover, neither Turkish immigrant children’s proficiency in either language nor their home language environment predicted EF. Our findings offer important new evidence in light of the ongoing debate about the existence of a bilingual advantage for EF.

## Introduction

The ability to speak two or more languages fluently offers its speakers manifold advantages such as communicative and intercultural benefits. For over a decade, research has claimed cognitive advantages for bilingual over monolingual speakers, specifically the reported benefit including higher levels of executive control in non-linguistic tasks [1, 2].

In the early 20th century, bilingualism was first understood as a detrimental circumstance to intellect and cognitive capacity of its speakers in the early 20^th^ century [3]. The research of Peal and Lambert [4] was a turning point providing evidence in favor of a bilingual advantage. However, it was Bialystok et al.’s [1] article that sparked a surge of research focused on determining the extent and conditions of a potential bilingual advantage for executive functions. More recently, however, replication studies [5, 6], as well as original research [7–9] refuting the claim of bilingual advantages on executive functions have become frequent.

## What are executive functions?

Executive functions (EF) are high-level cognitive abilities that require top-down control [10] and allow humans to show adaptive, goal-directed behavior in complex situations [11]. In an ever-changing and unpredictable environment, EFs are not only vital to successful performance in real-life situations but also associated with children’s potential to achieve academically [12, 13] and with psychological adjustment later in life (e.g., physical health and economic success; [14]). Executive functions are located in the prefrontal cortex [15] which is subject to prolonged postnatal development and thus highly susceptible to environmental influences during the first years of life. Accordingly, both genetic [16] and environmental factors contribute to individual EF differences [17, 18]. For example, it has been found that inhibition/attentional control is both genetically and environmentally influenced [19–23].

Research on the nature and interplay of different EF components, such as working memory and attentional control, has been complicated by conceptual and methodological differences across studies. One influential framework for the study of EFs has suggested three distinct but interrelated basic cognitive abilities as the core EF components: updating (also referred to as working memory), inhibition (attentional control), and shifting (cognitive flexibility) [24]. Working memory/updating refers to holding information in mind and manipulating it. Attentional control/inhibition refers to being able to focus one’s attention and suppress responses. Cognitive flexibility/shifting refers to being able to flexibly switch one’s attention and behavior back and forth between response sets and situational conditions. Because of operational difficulties to assess updating, inhibition, and shifting with separate tasks (i.e., task impurity), most EF researchers agree that a latent variable modeling approach is preferable in order to reliably measure different EF constructs and to extract the common variance that is shared among different tasks [24, 25]. Moreover, according to a recent review report [26], researchers today generally agree that EFs include working memory, inhibitory control, and cognitive flexibility, but there are still substantial differences between the theoretical models that propose to explain how EFs are specifically associated within neural, cognitive, and behavioral functions, e.g. see [26–28]. Finally, researchers have proposed models for how bilingualism may affect neural circuitry of executive control networks [29, 30], but the essential question of how bilinguals’ underlying neurocognitive processing may increase the efficiency of executive functions has not been resolved, in particular in relation to more general cognitive workload models [31, 32].

## What is the bilingual advantage?

Bilingual speakers develop two language systems either simultaneously or successively, and both language systems remain active when using either one of the languages [33, 34]. The theory behind this is that language use is monitored, and the non-relevant language is suppressed by the same general executive functions that are used to control attention and inhibition [33].

Several bilingual advantage hypotheses have been developed since the surge in research in this field. Originally, the *Inhibitory Control Advantage* hypothesis proposed that bilingual speakers, through extensive and prolonged exercise, are experts in consciously inhibiting the use of one or more of their languages while using another (also referred to as *Bilingual Inhibitory Control Advantage (BICA)*; [35]. This experience is believed to effectively train prefrontal executive control processes [36] which would then transfer to other dimensions of inhibitory control [37].

Potential effects of training the brain to monitor, suppress or switch language use also provide the theoretical foundation of newly emerging bilingual advantage hypotheses. The *Bilingual Executive Processing Advantage (BEPA)* hypothesis, for example, attributes overall faster response times for congruent, incongruent and neutral tasks to bilinguals’ abilities to resolve conflicts more efficiently [35]. The S*hifting Advantage* hypothesis refers to bilingual speakers’ ability to switch from one language to another with minimal switching cost, i.e., time [38].

The assumption that bilingual speakers benefit from cognitive advantages over monolinguals, particularly with regard to EF, has much support in language acquisition research [2]. In their seminal article, Bialystok et al. [1] reported significant advantages of bilingual over monolingual speakers in a series of three studies. The main finding was that older bilinguals responded significantly faster on the Simon task both with congruent and incongruent stimuli than their monolingual counterparts. Research has suggested that already at a very young age bilingual children show signs of better inhibitory control [39–43]. Executive functions are understood to benefit from an earlier onset of bilingualism [4, 44, 45] and develop to higher levels with bilingual experience [46]. With regard to socio-cultural background, the bilingual advantage has been found to be beneficial for children living in poverty [47, 48], and Bialystok and her colleagues conclude that bilinguals, regardless of their culture, outperform monolingual speakers [47, 49].

In recent years, criticism, particularly regarding methodological issues of studies investigating the bilingual advantage, has become more prevalent [5, 49, 50]. Shortcomings in the design and execution of research on the bilingual advantage have led to skepticism and calls for more scientific rigor including the focus on replication studies. Research on the bilingual advantage has generally been underpowered due to (very) small samples sizes [35, 50]. This is especially important to consider as the bilingual advantage has mainly been confirmed with smaller sample sizes. The larger the sample size, the lower the chance that significant results supporting the bilingual advantage were found [3, 35]. This suggests that previously reported group differences may have been false positive findings.

Furthermore, studies have either neglected to match groups for individual differences or have not included these potentially confounding variables in their statistical analyses. Few studies, for example, have statistically controlled for differences due to education and socio-economic status, immigration status or culture [50–53]. When studies have included individual difference variables, outcomes have often been inconsistent, e.g., for the age of onset and proficiency level [7–9, 54, 55] as well as for SES [45, 56]. In other instances, research outcomes supporting the bilingual advantage can plausibly be explained through differences in sampling, e.g., with regard to language proficiency or different cultural groups [51, 52]. For example, studies have reported that higher levels of language proficiency in both languages impact inhibitory control [48, 57]. Thus it should be accounted for.

Moreover, previous conclusions that the bilingual advantage is not confounded with socioeconomic and cultural factors due to matched designs may have been premature. For instance, Engel de Abreu et al. [47] compared Portuguese immigrant children in Luxembourg with monolingual children from Portugal. While the language background of these two groups may have been partly comparable, the same cannot be assumed for their general cultural background and socioeconomic living conditions, since Luxembourg and Portugal are characterized by vast cultural and sociodemographic differences. Thus, without a matched monolingual comparison group of children growing up in the same region we cannot conclude that potential bilingual advantages are not explained by other factors such as access to high-quality preschool education or stimulating home environments. Accordingly, one of the major concerns in the field is that replication studies have not been able to confirm the original findings [3, 5, 8, 35].

In their review of the available literature, Hilchey and Klein [35] conclude “that bilingual children never significantly outperformed monolingual children on inhibitory control, i.e., there was no evidence available to support a *Bilingual Inhibitory Control Advantage* [3, 35]. While Hilchey and Klein [35] previously reported a global RT advantage or BEPA for bilingual children, they did not report one in their updated review in 2015 [3], similarly, a very recent meta-analysis only shows very small effect sizes for BEPA [58].

Another highly relevant concern is the inconsistent definition and operationalization of bilingualism in the research literature. In this context, the assessment of proficiency in both languages should be determined by standardized, objective measures rather than, for example, proficiency self-assessments or years since the onset of bilingualism [45, 55, 59–61]. Many studies, however, either do not report [30, 56] or lack sufficient language testing for one or more of the languages of their participants. For example, a new US study by Hartanto, Toh and Yang [62] has recently confirmed bilingual advantages for inhibition and shifting in the large, nationally representative Early Childhood Longitudinal Study-Kindergarten (ECLS–K) sample. While these new results consistently confirm positive effects of higher socioeconomic status on various EF dimensions, reported effects of bilingualism are less uniform, depending on the predicted outcomes. What’s more, the group of bilinguals in this large dataset was composed of very heterogeneous languages and cultural backgrounds that are neither reported in detail nor controlled for in analyses, thus potentially attributing beneficial effects on children’s inhibition and shifting abilities to maternal reports of bilingualism without accounting for within-group variability. Nevertheless, Hartanto, Toh and Yang’s [62] results are noteworthy and important, and previous studies have made valid claims that the degree of bilingual language proficiency may positively affect children’s performance on EF measures [55, 59, 61].

Finally, a significant problem that may have distorted the overall understanding and trajectory of research in the field is significant publication bias. De Bruin et al. [63] showed that outcomes supporting the bilingual advantage were more likely to be published than negative results. Out of 104 conference abstracts included in their analysis, 68% of studies reporting a bilingual advantage were subsequently published in contrast to 29% of those without supporting evidence.

We aimed to address the shortcomings of previous studies by employing sophisticated structural equation modeling (SEM) analyses in a large sample of Turkish immigrant children in Germany and a control group of monolingual German children to test associations between bilingualism and EF. Latent variables in SEM allow for control of EF task impurity. We investigated the impact of language proficiency in both languages, i.e., Turkish and German tests, as well as children’s language environments on EF. The latter is particularly helpful to include as an environmental predictor as bilingualism can take various shapes in which the home language can be the immigrant language, the majority language, or a mixture of both languages. First, we tested if there were any differences between Turkish immigrant and German children’s EF scores, after controlling for parental education, child gender, age, and fluid intelligence (i.e., working memory). This analysis was carried out to replicate the design and results of previous studies that grouped children into monolingual and bilingual groups without assessing their actual language abilities. Thus, in line with previous studies that reported a bilingual advantage on EF, our first hypothesis was that Turkish immigrant children would do better than their monolingual German peers. Second, we hypothesized that a gradual increase in Turkish immigrant children’s bilingualism (e.g., higher scores in both languages, interaction effect of both languages) would be associated with better EF scores. This hypothesis is in line with the argument that bilingualism and the separation of languages enhance cognitive abilities, i.e., the BICA hypothesis [35], thus higher levels of language proficiency should lead to better EF performance. We would like to point out that we are not considering all Turkish immigrant children in our sample as bilingual, but instead we include their specific abilities in both Turkish and German as well as their exposure to languages as markers of gradual bilingualism.

## Methods

In accordance with local Faculty of Psychology Ethics Committee guidelines, ethical approval for data collection was obtained from the German Society for Psychology (DGPs), in addition, ethical approval for data analyses and publication was obtained from the University of Tennessee Institutional Review Board (UTK IRB-15-02562-XP).

### Participants and procedure

The SIMCUR project (Social Integration of Migrant Children—Uncovering Family and School Factors Promoting Resilience), is a cohort-sequential study on the development of Turkish immigrant children in Germany [64, 65]. In addition to Turkish immigrant families, non-immigrant German families were sampled for comparison purposes. Sampling took place in the Ruhr area, an industrial region in the North-West of Germany. Participants were screened via telephone by bilingual research assistants and considered eligible if the child’s mother or the mother’s parents or grandparents were born in Turkey. German participants were eligible if both parents were born in Germany and spoke German as their first language. In addition, in order to prevent confounding bias, children eligible for participation were born after 32 weeks of gestational age, were not living in a foster family, and did not have a referral to a special needs school (‘Sonderschule’). All parents that responded and fulfilled the inclusion criteria were included in the study. Families were allowed to choose if assessments took place in their homes or at the Ruhr-University in Bochum. During the first visit, mother interviews were conducted in German or Turkish by trained ethnically-matched native speakers, all structured interviews were available in Turkish and German. Confidentiality was explained, and parents and children signed consent forms. Families received €25 compensation. A total of *n* = 242 Turkish immigrant and *n* = 95 German children and adolescents (aged 5–15 years) participated in the current study. All immigrant children were of second or third generation (i.e., born in Germany; see Table 1). All children attended German schools where the language of instruction was German.

**Table 1:** Demographic and descriptive sample characteristics.

	Turkish immigrant sample, *n* = 242	German sample, *n* = 95
Child gender (*female*)	58.4%	49.5%
Age of child (*years;months*)	9;7 (2;11)	9;3 (3;1)
Digit span backwards score	5.07 (3.13)	5.57 (2.99)
Maternal education (ISCED)	1.84 (1.23)	3.23 (1.25)
Paternal education (ISCED)	2.22 (1.40)	3.01 (1.61)
Updating (efficiency, ms)	423 (149)	433 (186)
Inhibition (efficiency, ms)	530 (200)	567 (234)
Shifting (efficiency, ms)	713 (232)	820 (298)

Data is presented as *mean (SD)* for continuous variables and percentages *(%)* for categorical variables.

### Instruments

#### Parents’ education

Information on mothers’ and fathers’ vocational training and education (in Germany and Turkey) was obtained via interviews and coded according to the International Standard Classification of Education classification [66], ranging from primary (= 1) to tertiary (= 5) education. A new variable was created that reflected the highest ISCED code either mother or father had obtained. The ISCED codes have the advantage of providing a measure which allows comparisons between non-immigrant Germany and Turkish immigrant parents, who may have received education in Turkey as well as Germany.

#### Digit span backwards

As a marker of fluid intelligence, children were administered the digit span backwards task that asks to repeat increasingly longer strings of numbers presented aloud by the tester in reverse order. Successfully repeated strings were summed into a continuous score.

Bilingualism: Our analytical approach was informed by a contextual understanding of gradual bilingualism including children’s language proficiency (Turkish and German) and family environments (languages spoken with mother, father, and siblings), initially drawing from sociocultural models [67, 68] and ecological theories of human development [69, 70]. We operationalized this model as part of the SEM using vocabulary tests for Turkish and German, and detailed interviews about the amount of Turkish spoken at home, an indicator of children’s daily Turkish language use and exposure. Please note that all children in the sample attended German preschools and schools and thus they were exposed to German language environments on a daily basis.

#### Turkish vocabulary

Children’s receptive vocabulary in Turkish was used as a proxy for their proficiency and assessed with a specifically adapted, computer-based research version modeled on the Peabody Picture Vocabulary Test– 4^th^ edition (PPVT-4; [65, 71]).

#### German vocabulary

We used a German research version modeled on the English Expressive One-Word Picture Vocabulary Test (EOWPVT; [72, 73]).

In addition, we calculated an interaction term of Turkish*German proficiency to model bilingualism.

#### Family language use

Mothers provided information on language(s) they usually spoke with their children, as well as on the language(s) spoken with fathers and siblings. Responses were given on a 5-point Likert-type scale, with 1 = o*nly Turkish* and 5 = *only German*. Items were highly correlated and combined into a Turkish Family Environment Index score (reverse coded, higher score reflects higher Turkish language use).

Executive Functions: Children’s EFs were assessed with the computerized *Hearts and Flowers* task for E-Prime software and presented on laptops [74], computer keyboards were labeled with colored keys to ensure easy handling and appropriate reaction times that were not additionally confounded with motor and processing speed. The assessment included three separate conditions (i.e., blocks) [75], each representative of an EF component, which builds upon the previous condition and increases in difficulty and cognitive workload required. The first condition was congruent (i.e., hearts), and preceded by a training run before the actual data recording started. Children were instructed to press the computer key on the same side as the stimulus (i.e., heart) appeared on the screen. The congruent condition assessed updating abilities because children had to remember and apply a new rule. The second condition was incongruent (i.e., flowers), and also preceded by a training run. This time, children were asked to press the computer key on the opposite side from which the stimulus (i.e., flower) appeared on the screen. Thus, the incongruent condition required children to inhibit the predominant tendency to respond on the same side as the stimulus, as learned in the previous task. The third and final condition was mixed (i.e., both hearts and flowers combined), and assessed the child’s ability to flexibly shift from one rule to another. Thus, children needed to hold two abstract rules in mind (heart = ‘press same side,’ flower = ‘press opposite side’), inhibit their predominant responses for incongruent items and use cognitive flexibility to switch back and forth between congruent and incongruent response modes. Response time in milliseconds (ms) and accuracy were recorded for each item. For main analyses, responses for congruent, incongruent, and mixed EF task conditions were combined to load onto one latent EF variable using efficiency scores (i.e., each participant’s median response times on correct items in each condition). Thus, the latent EF variable represents the shared variance among the three observed, manifest variables, congruent, incongruent, and mixed, respectively. Please note that for our outcome variables, lower scores, i.e., reaction times, indicate better EF scores.

### Statistical analyses

Analyses were performed in SPSS 24 and AMOS 24. Two-sided significance levels were set to 5%. All continuous scores were z-standardized and analyses controlled for child gender, age, and parents’ level of education, digit span backwards was added as a mediator. We estimated two structural equation models. In model 1, Turkish immigrant and monolingual German children’s executive functions were compared. In model 2, only Turkish immigrant children were included, and the extent of their specific bilingual environments and abilities was estimated.

## Results

Table 1 provides demographic information about the Turkish immigrant and German participants.

**Fig 1:**
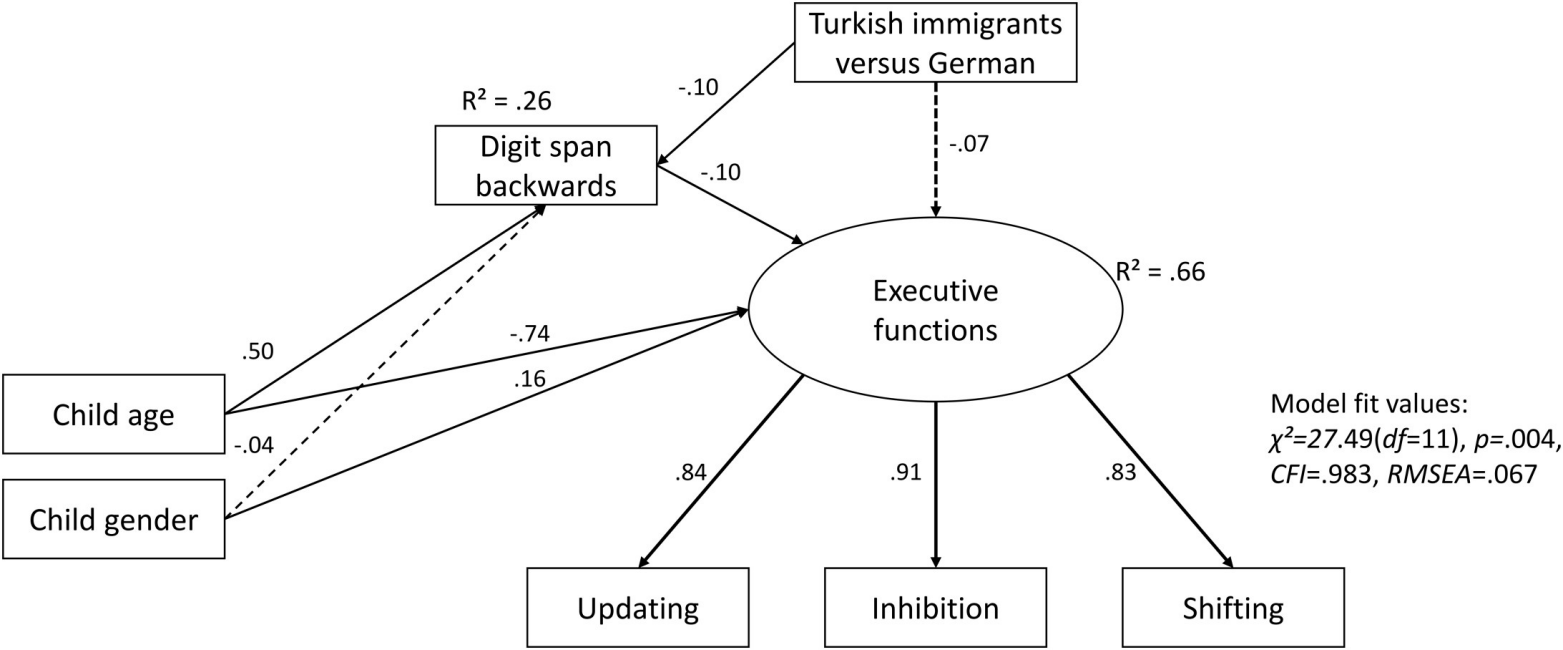
Structural equation model 1 (SEM) of Turkish immigrant and German monolingual, non-immigrant children‘s (N = 337) executive functions. Please note: for simplicity, error terms are not shown.

In SEM Model 1, Turkish immigrant and monolingual German children’s executive functions were compared. Parental education was not related to EF abilities and was removed from the model for reasons of parsimony and statistical fit. While model fit was acceptable (*χ*^*2*^ = 27.49(*df* = 13), *p* = .004, *CFI* = .983, *RMSEA* = .067, with 66% of variance explained in EF (*R*^*2*^)), path estimates showed that Turkish immigrant and German children’s EF skills did not significantly differ (*ß* = -.07, *p* = .055), after controlling for child gender and age (Fig 1). Turkish immigrant children had lower digit span backwards scores than their German peers (*ß* = -.10, *p* = .043), while higher digit span backwards scores were associated with better EF abilities in both groups (*ß* = -.10, *p* = .020; i.e., lower efficiency scores).

**Fig 2:**
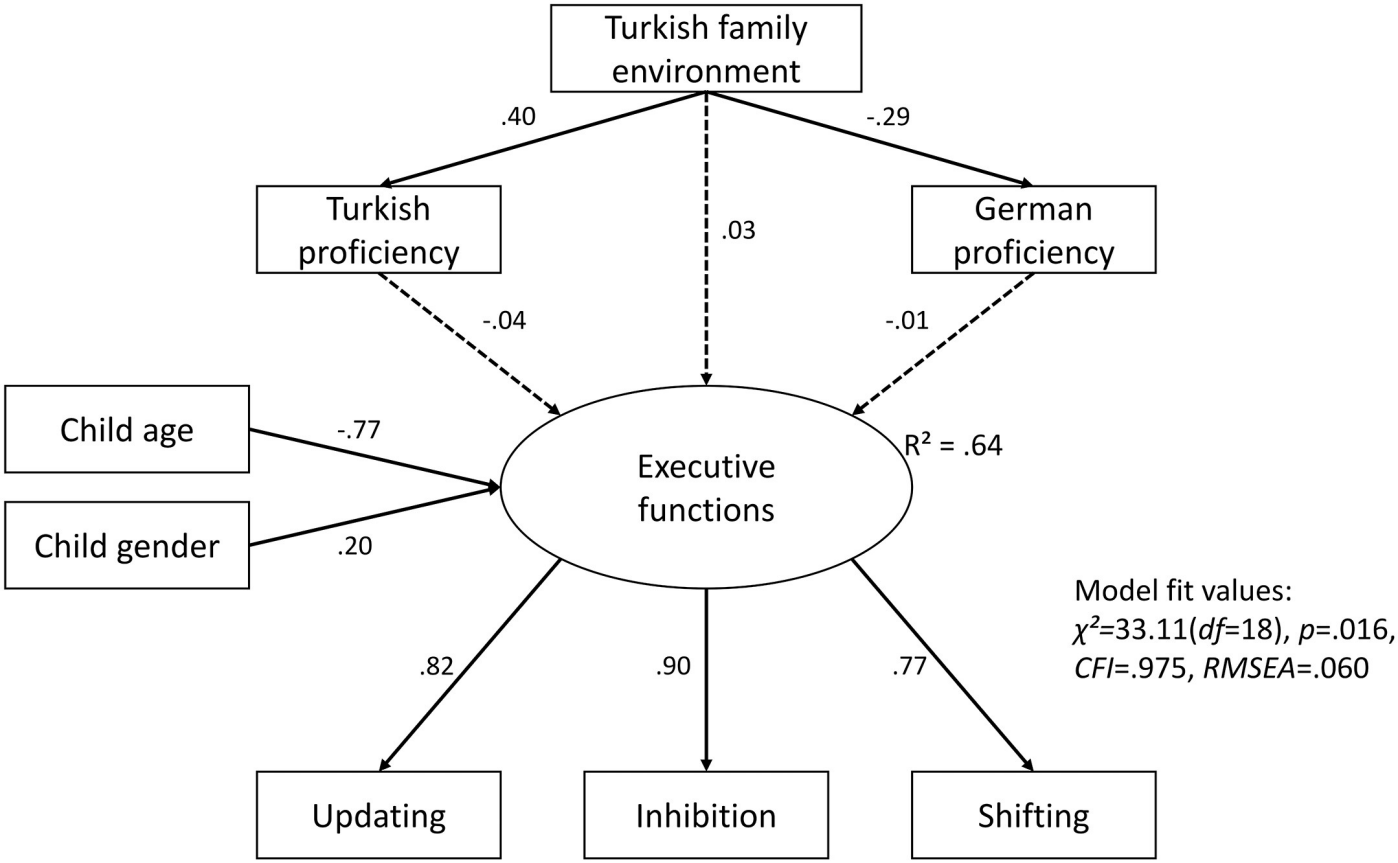
Structural equation model 2 testing effects of Turkish immigrant children’s (N = 242) bilingual language environment and proficiency on executive functions. Please note: for simplicity, error terms are not shown.

In Model 2, only Turkish immigrant children were included, and their specific bilingual environments and proficiency were included to test if higher levels of Turkish and German vocabulary knowledge, as well as higher exposure to Turkish at home, were associated with better EF skills. As before, parental education was not related to EF abilities and was removed from the model. In addition, in light of the smaller sample size in this second model, digit span backwards scores were removed from the model, for reasons of parsimony and since their effects were not part of the main hypotheses (Fig 2). While both gender (*ß* = .20, *p* < .001) and age (*ß* = -.77, *p* < .001) significantly predicted children’s EF abilities, neither Turkish (*ß* = -.04, *p* = .374) nor German (*ß* = -.01, *p* = .838) language proficiency nor the amount of Turkish spoken at home (*ß* = .03, *p* = -.593) predicted EF. Moreover, we also tried to include an interaction term of Turkish*German proficiency to model bilingualism, but this term was not significant and reduced model fit. We thus excluded it for reasons of statistical parsimony. Despite the hypothesis-driven inclusion of non-significant paths in the specified model, fit values were acceptable: *χ*^*2*^ = 33.11 (*df* = 18), *p* = .016, *CFI* = .975, *RMSEA* = .060, with 64% of variance explained in EF (*R*^*2*^).

Finally, we removed all language indicators from the SEM to validate our findings, included both Turkish and German participants (N = 337), and obtained excellent model fit values (*χ*^*2*^ = 2.15*(df* = 5), *p* = .828, *CFI* = 1.00, *RMSEA* = .00, 65% variance explained in EF (*R*^*2*^)).

## Discussion

Our results support the growing number of scientifically rigorous studies that have failed to replicate the bilingual EF advantage [6, 7, 50]. The current SEM analyses on large samples of 5 to 15 years old participants do not show a significant bilingual advantage among Turkish immigrant or German children’s EFs. Further, neither language proficiency in Turkish and German nor the Turkish family environment resulted in a significant association with immigrant children’s EF. In our models, child age and gender were the only variables that predicted EF. In both models, we also controlled for parents’ educational backgrounds, but we found no evidence of parents’ education being connected to children’s EF. While the lack of a significant effect of parents’ education as a proxy for the socio-economic status in our analyses was surprising [45], we excluded the variable from the final models for statistical reasons. It is well known in the cross-cultural literature that immigrants’ levels of completed education does not necessarily reflect their actual academic abilities, because they may not have had sufficient access to education in their home countries, and because their academic careers may have been disrupted by the migration process [68, 76, 77]. Thus, the effect of parents’ education on their children’s EF abilities may have been lower than in other non-immigrant samples in the general EF and cognitive development literature [18, 78]. This suggests that for this immigrant sample educational background may be less closely associated with EF than usually found.

In addition, the inconsistent use of different tests methods and various selections of EF components as outcome variables, such as assessing only inhibition or shifting, or assessing only accuracy without timing, or reaction times on both correct and wrong items, hinder proper comparisons between studies. The use of different test task constellations and environmental conditions also needs to be viewed in the context of nuanced differences between scientific fields with regard to their understandings of (a) the construct of EF and (b) the bilingual advantage [35].

In the present study, EFs were assessed via test items tapping into updating, inhibition, and shifting that were combined into a latent variable construct. While each of these components can be viewed as requiring unique cognitive skills that may independently profit from bilingual abilities and enable better everyday functioning, they can hardly be assessed independently of each other (i.e., task impurity) and are indeed highly correlated. The high loadings of each test component onto the latent variable EF, and the excellent fit values of the final, reduced model that mainly reflect the fit of the confirmatory factor analysis (CFA) for EF, are proof of our approach’s validity and reliability.

Higher fluid intelligence (i.e., digit span backwards) scores were associated with better EF abilities. Interestingly, while Turkish immigrant children had lower digit span scores, there was a simultaneous non-significant trend towards better EF scores, compared to their German peers. Although our results failed to replicate a bilingual advantage for EF, these trends suggest that there is merit in further investigating the neurodevelopmental mechanisms underlying potential bilingual advantages across specific cognitive functions.

Although our findings add to recent studies that have failed to replicate the bilingual advantage for EF, potential other benefits of being competent in more than one language should continue to be investigated in future studies. From a pedagogical viewpoint, it is also important to understand under which circumstances participants have acquired (i.e., family languages or immersion through migration or school) or learned (i.e., foreign language classes) spoken language, and how they developed their reading and writing skills [79]. Acquiring a language in an informal environment, for example, will help develop basic interpersonal communication skills (BICS), while it may not foster higher-level literacy skills that include cognitive academic language proficiency (CALP).

Our results are based on a large sample of Turkish immigrant children, however, it may be argued that merely considering *p* values when interpreting statistical results is not sufficient, partly because statistical significance is highly correlated with sample size. This is why we applied SEM and considered a number of additional model fit values. Moreover, the risk for false-positive as well as false-negative findings generally decreases with increased sample sizes, while we have investigated the size of a contextualized gradual effect of bilingualism on EF performance.

We acknowledge that our approach to modeling children’s bilingualism, including their language proficiency and language environments, merely represents a simplified account of the actual daily interactions and exposures that shape their bilingual skills and identities. Nevertheless, we chose this approach over just grouping children into bilingual versus monolingual. Further, a limitation in the present study and in the field, in general, is the lack of accounting for effects of biliteracy when considering bilingualism. Research on the acquisition of languages, for example, has shown that the ability to read and write in one’s L1 and L2 positively impacts learning or acquiring an additional language [80–82]. Developing biliteracy requires a certain level of deliberate language planning by parents and circumstances under which the immigrant or non-majority language is offered through school or community-based courses. While assessing these skills is time-consuming, the potential of these written bilingual abilities should not be underestimated [80], particularly in the context of explaining potential cognitive benefits from being bilingual or biliterate.

## Conclusion

Taken together, our findings add strong support to recently accumulating evidence that the bilingual advantage cannot be replicated. Our sophisticated analyses on a large sample did not show any effects of immigrant or bilingual background on EF. Previous studies that claimed significant bilingual advantages on EF may have over-estimated group differences, potentially due to false-positive findings, sampling bias, or failure to control for confounders. Large, well-designed, objective and rigorous studies that assess specific aspects of bilingualism in the context of demographic and environmental characteristics are needed to inform the ongoing scientific debate. Nevertheless, the ability to speak two languages without a doubt has the potential for significant advantages for individuals and societies, reaching far beyond the linguistic realm.

## References

[1] Bialystok E, Craik FIM, Klein R, Viswanathan M. Bilingualism, aging, and cognitive control: Evidence from the Simon task. Psychol Aging 2004; 19(2):290–303. 10.1037/0882-7974.19.2.290 15222822

[2] Bialystok E. Bilingualism and the Development of Executive Function: The Role of Attention. Child Dev Perspect 2015; 9(2):117–21. 10.1111/cdep.12116 26019718 PMC4442091

[3] Hilchey MO, Saint-Aubin J, Klein RM. Does bilingual exercise enhance cognitive fitness in traditional non-linguistic executive processing tasks? In: Schwieter JW, editor. The Cambridge Handbook of Bilingual Processing. Cambridge: Cambridge University Press; 2015 p. 586–613.

[4] Pelham SD, Abrams L. Cognitive advantages and disadvantages in early and late bilinguals. J Exp Psychol Learn Mem Cogn 2014; 40(2):313–25. 10.1037/a0035224 24294916

[5] Paap KR, Greenberg ZI. There is no coherent evidence for a bilingual advantage in executive processing. Cogn Psychol 2013; 66(2):232–58. 10.1016/j.cogpsych.2012.12.002 23370226

[6] Kirk NW, Fiala L, Scott-Brown KC, Kempe V. No evidence for reduced Simon cost in elderly bilinguals and bidialectals. J Cogn Psychol (Hove) 2014; 26(6):640–8. 10.1080/20445911.2014.929580 25264481 PMC4164011

[7] Kousaie S, Phillips NA. Ageing and bilingualism: Absence of a “bilingual advantage” in stroop interference in a nonimmigrant sample. Q J Exp Psychol (Hove) 2012; 65(2):356–69. 10.1080/17470218.2011.604788 21936646

[8] Morton JB, Harper SN. What did Simon say? Revisiting the bilingual advantage. Dev Sci 2007; 10(6):719–26. 10.1111/j.1467-7687.2007.00623.x 17973787

[9] von Bastian CC, Souza AS, Gade M. No evidence for bilingual cognitive advantages: A test of four hypotheses. J Exp Psychol Gen 2016; 145(2):246–58. 10.1037/xge0000120 26523426

[10] Diamond A. Executive functions. Annu Rev Psychol 2013; 64:135–68. 10.1146/annurev-psych-113011-143750 23020641 PMC4084861

[11] Garon N, Bryson SE, Smith IM. Executive function in preschoolers: A review using an integrative framework. Psychol Bull 2008; 134(1):31–60. 10.1037/0033-2909.134.1.31 18193994

[12] Best JR, Miller PH, Naglieri JA. Relations between Executive Function and Academic Achievement from Ages 5 to 17 in a Large, Representative National Sample. Learn Individ Differ 2011; 21(4):327–36. 10.1016/j.lindif.2011.01.007 21845021 PMC3155246

[13] Bull R, Espy KA, Wiebe SA. Short-term memory, working memory, and executive functioning in preschoolers: Longitudinal predictors of mathematical achievement at age 7 years. Dev Neuropsychol 2008; 33(3):205–28. 10.1080/87565640801982312 18473197 PMC2729141

[14] Moffitt TE, Arseneault L, Belsky D, Dickson N, Hancox RJ, Harrington H et al A gradient of childhood self-control predicts health, wealth, and public safety. Proc Natl Acad Sci U S A 2011; 108(7):2693–8. 10.1073/pnas.1010076108 21262822 PMC3041102

[15] Jurado MB, Rosselli M. The elusive nature of executive functions: A review of our current understanding. Neuropsychol Rev 2007; 17(3):213–33. 10.1007/s11065-007-9040-z 17786559

[16] Friedman NP, Miyake A, Young SE, Defries JC, Corley RP, Hewitt JK. Individual differences in executive functions are almost entirely genetic in origin. J Exp Psychol Gen 2008; 137(2):201–25. 10.1037/0096-3445.137.2.201 18473654 PMC2762790

[17] Braver TS, Cole MW, Yarkoni T. Vive les differences! Individual variation in neural mechanisms of executive control. Curr Opin Neurobiol 2010; 20(2):242–50. 10.1016/j.conb.2010.03.002 20381337 PMC2904672

[18] Hughes C. Changes and challenges in 20 years of research into the development of executive functions. Inf. Child Develop. 2011; 20(3):251–71. 10.1002/icd.736

[19] Farah MJ, Shera DM, Savage JH, Betancourt L, Giannetta JM, Brodsky NL et al Childhood poverty: Specific associations with neurocognitive development. Brain Res 2006; 1110(1):166–74. 10.1016/j.brainres.2006.06.072 16879809

[20] Gagne JR, Saudino KJ. Wait for it! A twin study of inhibitory control in early childhood. Behav Genet 2010; 40(3):327–37. 10.1007/s10519-009-9316-6 19936910 PMC2854273

[21] Noble KG, Houston SM, Kan E, Sowell ER. Neural correlates of socioeconomic status in the developing human brain. Dev Sci 2012; 15(4):516–27. 10.1111/j.1467-7687.2012.01147.x 22709401 PMC6554027

[22] Polderman TJC, Huizink AC, Verhulst FC, van Beijsterveldt CEM, Boomsma DI, Bartels M. A genetic study on attention problems and academic skills: Results of a longitudinal study in twins. J Can Acad Child Adolesc Psychiatry 2011; 20(1):22–34. 21286366 PMC3024720

[23] Rueda MR, Rothbart MK, McCandliss BD, Saccomanno L, Posner MI. Training, maturation, and genetic influences on the development of executive attention. Proc Natl Acad Sci U S A 2005; 102(41):14931–6. 10.1073/pnas.0506897102 16192352 PMC1253585

[24] Miyake A, Friedman NP. The Nature and Organization of Individual Differences in Executive Functions: Four General Conclusions. Curr Dir Psychol Sci 2012; 21(1):8–14. 10.1177/0963721411429458 22773897 PMC3388901

[25] Zelazo PD, Blair CB, Willoughby MT. Executive Function: Implications for Education. Washington, DC: National Center for Education Research, Institute of Education Sciences; 2016 [cited 2018 Sep 18]. URL: https://ies.ed.gov/ncer/pubs/20172000/.

[26] Zelazo PD. Executive function: Reflection, iterative reprocessing, complexity, and the developing brain. Developmental Review 2015; 38:55–68. 10.1016/j.dr.2015.07.001

[27] Blair C, Raver CC. School readiness and self-regulation: a developmental psychobiological approach. Annu Rev Psychol 2015; 66:711–31. 10.1146/annurev-psych-010814-015221 25148852 PMC4682347

[28] Buss AT, Spencer JP. The emergent executive: a dynamic field theory of the development of executive function. Monogr Soc Res Child Dev 2014; 79(2):vii, 1–103. 10.1002/mono.12096 24818836 PMC4426851

[29] Costa A, Santesteban M. Lexical access in bilingual speech production: Evidence from language switching in highly proficient bilinguals and L2 learners. Journal of Memory and Language 2004; 50(4):491–511. 10.1016/j.jml.2004.02.002

[30] Hernández M, Costa A, Fuentes LJ, Vivas ABNA, Sebastián-Gallés N. The impact of bilingualism on the executive control and orienting networks of attention. Bilingualism 2010; 13(03):315–25. 10.1017/S1366728909990010

[31] Jaekel J, Baumann N, Wolke D. Effects of gestational age at birth on cognitive performance: a function of cognitive workload demands. PLoS ONE 2013; 8(5):e65219 10.1371/journal.pone.0065219 23717694 PMC3663809

[32] Just MA, Varma S. The organization of thinking: What functional brain imaging reveals about the neuroarchitecture of complex cognition. Cognitive, Affective, & Behavioral Neuroscience 2007; 7(3):153–91. 10.3758/cabn.7.3.153 17993204

[33] Green DW. Mental control of the bilingual lexico-semantic system. Bilingualism 1998; 1(02):67 10.1017/S1366728998000133

[34] van Heuven WJB, Schriefers H, Dijkstra T, Hagoort P. Language conflict in the bilingual brain. Cereb Cortex 2008; 18(11):2706–16. 10.1093/cercor/bhn030 18424776 PMC2567421

[35] Hilchey MD, Klein RM. Are there bilingual advantages on nonlinguistic interference tasks? Implications for the plasticity of executive control processes. Psychon Bull Rev 2011; 18(4):625–58. 10.3758/s13423-011-0116-7 21674283

[36] Ashcraft MH, Klein RM. Cognition. Canadian ed Toronto: Pearson Canada; 2009.

[37] Bialystok E. Bilingualism in development: Language, literacy, and cognition. Cambridge: Cambridge University Press; 2001.

[38] Bialystok E, Craik FIM, Luk G. Bilingualism: Consequences for mind and brain. Trends Cogn Sci (Regul Ed) 2012; 16(4):240–50. 10.1016/j.tics.2012.03.001 22464592 PMC3322418

[39] Bialystok E. Cognitive Complexity and Attentional Control in the Bilingual Mind. Child Dev 1999; 70(3):636–44. 10.1111/1467-8624.00046

[40] Bialystok E, Martin MM. Attention and inhibition in bilingual children: Evidence from the dimensional change card sort task. Dev Sci 2004; 7(3):325–39. 10.1111/j.1467-7687.2004.00351.x 15595373

[41] Poulin-Dubois D, Blaye A, Coutya J, Bialystok E. The effects of bilingualism on toddlers’ executive functioning. J Exp Child Psychol 2011; 108(3):567–79. 10.1016/j.jecp.2010.10.009 21122877 PMC4346342

[42] Bialystok E. How does experience change cognition? Evaluating the evidence. Br J Psychol 2011; 102(3):303–5; discussion 309–12. 10.1111/j.2044-8295.2011.02008.x 21751988

[43] Filippi R, Morris J, Richardson FM, Bright P, Thomas MSC, Karmiloff-Smith A et al Bilingual children show an advantage in controlling verbal interference during spoken language comprehension. Bilingualism 2015; 18(3):490–501. 10.1017/S1366728914000686 26146479 PMC4486347

[44] Luk G, Anderson JAE, Craik FIM, Grady C, Bialystok E. Distinct neural correlates for two types of inhibition in bilinguals: Response inhibition versus interference suppression. Brain Cogn 2010; 74(3):347–57. 10.1016/j.bandc.2010.09.004 20965635

[45] Carlson SM, Meltzoff AN. Bilingual experience and executive functioning in young children. Dev Sci 2008; 11(2):282–98. 10.1111/j.1467-7687.2008.00675.x 18333982 PMC3647884

[46] Luk G, de Sa E, Bialystok E. Is there a relation between onset age of bilingualism and enhancement of cognitive control? Bilingualism 2011; 14(04):588–95. 10.1017/S1366728911000010

[47] Engel de Abreu PMJ, Cruz-Santos A, Tourinho CJ, Martin R, Bialystok E. Bilingualism enriches the poor: Enhanced cognitive control in low-income minority children. Psychol Sci 2012; 23(11):1364–71. 10.1177/0956797612443836 23044796 PMC4070309

[48] Ladas AI, Carroll DJ, Vivas AB. Attentional processes in low-socioeconomic status bilingual children: Are they modulated by the amount of bilingual experience? Child Dev 2015; 86(2):557–78. 10.1111/cdev.12332 25571905

[49] Bialystok E, Viswanathan M. Components of executive control with advantages for bilingual children in two cultures. Cognition 2009; 112(3):494–500. 10.1016/j.cognition.2009.06.014 19615674 PMC2755257

[50] Paap KR, Johnson HA, Sawi O. Bilingual advantages in executive functioning either do not exist or are restricted to very specific and undetermined circumstances. Cortex 2015; 69:265–78. 10.1016/j.cortex.2015.04.014 26048659

[51] Morton JB. Still waiting for real answers. Cortex 2015; 73:352–3. 10.1016/j.cortex.2015.07.010 26298268

[52] Klein RM. On the belief that the cognitive exercise associated with the acquisition of a second language enhances extra-linguistic cognitive functions: Is “Type-I incompetence” at work here? Cortex 2015; 73:340–1. 10.1016/j.cortex.2015.07.020 26298267

[53] Costa A, Hernández M, Sebastián-Gallés N. Bilingualism aids conflict resolution: evidence from the ANT task. Cognition 2008; 106(1):59–86. 10.1016/j.cognition.2006.12.013 17275801

[54] Paap KR, Johnson HA, Sawi O. Are bilingual advantages dependent upon specific tasks or specific bilingual experiences? J Cogn Psychol (Hove) 2014; 26(6):615–39. 10.1080/20445911.2014.944914

[55] Costa A, Santesteban M, Ivanova I. How do highly proficient bilinguals control their lexicalization process? Inhibitory and language-specific selection mechanisms are both functional. J Exp Psychol Learn Mem Cogn 2006; 32(5):1057–74. 10.1037/0278-7393.32.5.1057 16938046

[56] Antón E, Duñabeitia JA, Estévez A, Hernández JA, Castillo A, Fuentes LJ et al Is there a bilingual advantage in the ANT task? Evidence from children. Front Psychol 2014; 5:398 10.3389/fpsyg.2014.00398 24847298 PMC4019868

[57] Chen SH, Zhou Q, Uchikoshi Y, Bunge SA. Variations on the bilingual advantage? Links of Chinese and English proficiency to Chinese American children’s self-regulation. Front Psychol 2014; 5:1069 10.3389/fpsyg.2014.01069 25324795 PMC4179764

[58] Paap KR. The Bilingual Advantage Debate: Quantity and Quality of the Evidence In: Schwieter JW, editor. The handbook of the neuroscience of multilingualism. First edition Hoboken, NJ: Wiley-Blackwell; 2019 p. 701–35 [The handbook of childhood language].

[59] Blom E, Küntay AC, Messer M, Verhagen J, Leseman P. The benefits of being bilingual: Working memory in bilingual Turkish-Dutch children. J Exp Child Psychol 2014; 128:105–19. 10.1016/j.jecp.2014.06.007 25160938

[60] Verhagen J, Mulder H, Leseman PPM. Effects of home language environment on inhibitory control in bilingual three-year-old children. Bilingualism 2017; 20(01):114–27. 10.1017/S1366728915000590

[61] Poarch GJ, van Hell JG. Executive functions and inhibitory control in multilingual children: evidence from second-language learners, bilinguals, and trilinguals. J Exp Child Psychol 2012; 113(4):535–51. 10.1016/j.jecp.2012.06.013 22892367

[62] Hartanto A, Toh WX, Yang H. Bilingualism Narrows Socioeconomic Disparities in Executive Functions and Self-Regulatory Behaviors During Early Childhood: Evidence From the Early Childhood Longitudinal Study. Child Dev 2018 10.1111/cdev.13032 29318589

[63] de Bruin A, Della Sala S. The decline effect: How initially strong results tend to decrease over time. Cortex 2015; 73:375–7. 10.1016/j.cortex.2015.05.025 26093779

[64] Jaekel J, Leyendecker B, Agache A. Family and Individual Factors Associated with Turkish Immigrant and German Children’s and Adolescents’ Mental Health. J Child Fam Stud 2015; 24(4):1097–105. 10.1007/s10826-014-9918-3

[65] Willard JA, Agache A, Jaekel J, Glück CW, Leyendecker B. Family factors predicting vocabulary in Turkish as a heritage language. Applied Psycholinguistics 2015; 36(04):875–98. 10.1017/S0142716413000544

[66] UNESCO. ISCED 1997: International Standard Classification of Education; 2006.

[67] García Coll C., Pachter LM. Ethnic and minority parenting In: Bornstein MH, editor. Social conditions and applied parenting. 2^nd^ ed Mahwah, N.J.: Erlbaum; 2002 [Handbook of parenting; v. 4].

[68] Leyendecker B. Die frühe Kindheit in Migrantenfamilien In: Keller H, editor. Handbuch der Kleinkindforschung. 3. Aufl. Bern: Hans Huber; 2003 p. 381–431.

[69] Bronfenbrenner U. Toward an experimental ecology of human development. American Psychologist 1977; 32(7):513–31. 10.1037/0003-066X.32.7.513

[70] Hornberger NH. Multilingual language policies and the continua of biliteracy: An ecological approach. Language Policy 2002; 1(1):27–51. 10.1023/a:1014548611951

[71] Glück CW. Receptive Vocabulary Test research version modeled on PPVT-4th edition for NUBBEK [Unpublished manuscript]; 2009.

[72] Brownell R. Expressive One-Wor Picture Vocabulary Test (EOWPVT). Novato, CA: Academic Therapy Publications; 2000.

[73] Martin N., Brownell R. Expressive One Word Picture Vocabulary Test—Forth Edition (EOWPVT-IV). Austin, TX: Pro-ED; 2010.

[74] Wright A, Diamond A. An effect of inhibitory load in children while keeping working memory load constant. Front Psychol 2014; 5:213 10.3389/fpsyg.2014.00213 24672502 PMC3954128

[75] Davidson MC, Amso D, Anderson LC, Diamond A. Development of cognitive control and executive functions from 4 to 13 years: Evidence from manipulations of memory, inhibition, and task switching. Neuropsychologia 2006; 44(11):2037–78. 10.1016/j.neuropsychologia.2006.02.006 16580701 PMC1513793

[76] Jaekel J, Leyendecker B. Tägliche Stressfaktoren und Lebenszufriedenheit türkischstämmiger Mütter in Deutschland. Zeitschrift für Gesundheitspsychologie 2008; 16(1):12–21. 10.1026/0943-8149.16.1.12

[77] Leyendecker B, Schölmerich A, Citlak B. Similarities and differences between first- and second-generation Turkish migrant mothers in Germany: The acculturation gap In: Bornstein MH, Cote LR, editors. Acculturation and parent-child relationships: Measurement and development. Mahwah, N.J., London: Lawrence Erlbaum Associates; 2006 p. 297–315 [Monographs in parenting series].

[78] Bornstein MH, Hahn C-S, Wolke D. Systems and cascades in cognitive development and academic achievement. Child Dev 2013; 84(1):154–62. 10.1111/j.1467-8624.2012.01849.x 22974268 PMC3525805

[79] Jaekel N, Schurig M, Florian M, Ritter M. From Early Starters to Late Finishers?: A Longitudinal Study of Early Foreign Language Learning in School. Language Learning 2017; 67(3):631–64. 10.1111/lang.12242

[80] Rauch DP, Naumann J, Jude N. Metalinguistic awareness mediates effects of full biliteracy on third-language reading proficiency in Turkish–German bilinguals. International Journal of Bilingualism 2011; 16(4):402–18. 10.1177/1367006911425819

[81] Cummins J. Linguistic Interdependence and the Educational Development of Bilingual Children. Review of Educational Research 1979; 49(2):222 10.2307/1169960

[82] Cummins J. Language, power and pedagogy: Bilingual children in the crossfire. Clevedon: Multilingual Matters; 2000. (Bilingual education and bilingualism; vol 23).

